# Stability analysis of SEIR model related to efficiency of vaccines for COVID-19 situation

**DOI:** 10.1016/j.heliyon.2021.e06812

**Published:** 2021-04-15

**Authors:** Phitchayapak Wintachai, Kiattisak Prathom

**Affiliations:** a Division of Biology, School of Science, Walailak University, Nakhon Si Thammarat, Thailand; b Division of Mathematics and Statistics, School of Science, Walailak University, Nakhon Si Thammarat, Thailand

**Keywords:** COVID-19, Vaccination, SEIR model, Stability, Prophylactic, Therapeutic

## Abstract

This work is aimed to formulate and analyze a mathematical modeling, SEIR model, for COVID-19 with the main parameters of vaccination rate, effectiveness of prophylactic and therapeutic vaccines. Global and local stability of the model are investigated and also numerical simulation. Local stability of equilibrium points are classified. A Lyapunov function is constructed to analyze global stability of the disease-free equilibrium. The simulation part is based on two situations, the US and India. In the US circumstance, the result shows that with the rate of vaccination 0.1% per day of the US population and at least 20% effectiveness of both prophylactic and therapeutic vaccines, the reproductive numbers $R_{0}$ are reduced from 2.99 (no vaccine) to less than 1. The same result happens in India case where the maximum reproductive number $R_{0}$ in this case is 3.38. To achieve the same infected level of both countries, the simulation shows that with the same vaccine's efficiency the US needs a higher vaccination rate per day. Without vaccines for this pandemic, the model shows that a few percentages of the populations will suffering from the disease in the long term.

## 1 Introduction

Coronavirus disease is a severe acute respiratory disease caused by a coronavirus 2 (SARS-CoV-2) that is a new member of the genus *Beta coronavirus* and family *Coronaviridae* [1], [2]. The virus primarily spreads from person to person through droplet, airborne, and contact transmission. The clinical symptoms of SARS-CoV-2 infected patients had mild, moderate, and severe symptoms such as fever, dry cough, difficulty breathing, fatigue, new loss of taste or smell, nausea, diarrhea, pneumonia, and respiratory symptom [3], [4]. The critically severe conditions such as chronic medical illness, organ dysfunctions, and death have been frequently reported in elderly patients and people with immunodeficiencies [5], [6]. However, many SARS-CoV-2 infected patients are minimally symptomatic or asymptomatic [7], [8], [9].

The outbreak of SARS-CoV-2 started in China and then transmitted to humans and animals [10], [11]. Nowadays, the virus has recently caused epidemics around the world in more than 215 countries with 46,403,652 confirmed cases and 1,198,569 mortalities, as of November 2, 2020 [12]. According to a recent report from the World Health Organization on SARS-CoV-2 outbreak, the number of confirmed cases in America is higher than the number of confirmed cases in Europe, South-East-Asia, Eastern Mediterranean, Africa, and Western Pacific, respectively. The top two countries reporting the most confirmed cases are the United States of America (9,032,465 cases) and India (8,229,313 cases). As COVID-19 cases keep increasing, predictions of the number of infected cases and the termination of COVID-19 are worth it to study. Mathematical model of infectious diseases is a crucial tool that has been used to study dynamics of how diseases spread. A mathematical model can predict the future situation of an outbreak and evaluate the best strategy to control spreading diseases. There are many different types of mathematical models for predicting an epidemic infection. One of them is called compartment models.

Compartment model is an interesting tool for COVID-19 situation. It is a powerful mathematical model for understanding the complex dynamics of epidemics. In this work we construct a well known model called SEIR model with 4 compartments of susceptible population *S*, exposed population *E*, infectious population *I*, and recovered population *R*. The model SEIR is suitable for disease transmission which an infected individual needs a short time period to be an infectious. Many researches have been studied by adapting SEIR model to forecast dynamics of endemic and epidemic such as Dengue Fever [13], [14], [15], Ebola [16], [17], Middle East Respiratory Syndrome (MERS) [18], [19], Severe Acute Respiratory Syndrome (SARS) [20], [21], to name a few. According to [22], [23], [24], COVID-19 has an average incubation period of 11.5 days before spreading of the viruses, so SEIR model is suitable for predicting COVID-19 situation. SEIR model have been adapted by adding strategy parameters such as social distancing and face mask using to control and predict COVID-19 situation in several researches [25], [26], [27], [28], [29], [30].

Vaccine administration is a highly effective method of preventing and reducing viral infections [31]. Even though there is no vaccine or a specific antiviral for the treatment of patients infected with SARS-CoV-2 available, several vaccines against SARS-CoV-2 such as a live attenuated vaccine, inactivated vaccines, subunit vaccines, DNA and RNA vaccines, and vector vaccines have been developed [32], [33]. Vaccination and optimal control are key points to control an epidemic situation as discussed in [34], [35], [36], [37]. In this study, we use SEIR model equipped with effectiveness of vaccination to forecast COVID-19 situation when a vaccine comes out. There are two main types of vaccine in our SEIR model prophylactic and therapeutic vaccines. Prophylactic vaccine is a preventing vaccine and therapeutic vaccine is a vaccine that is administrated after infection [38].

In Section 2, we formulate a model and investigate all equilibrium points together with their global and local stability of the model. Section 3 is mathematical simulation part. In particular, we applied recorded parameters of US and India circumstances to our model and predicted the potential of COVID-19 in both countries when vaccines come out.

## 2 Model formulation

**Figure 1:**
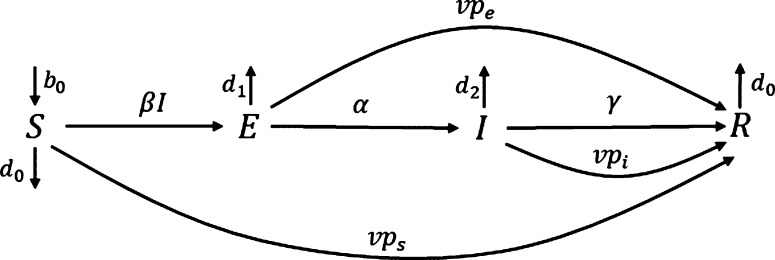
Schematic diagram of *SEIR* model for COVID-19 situation.

We consider the 4-compartment model called SEIR which S(t),E(t),I(t), and R(t) are the fractions of susceptible, exposed, infectious, and recovered populations, respectively, at the time *t*. The trivial solution S≡0, E≡0, I≡0 and R≡0 is out of our interest. The system of differential equations related to the schematic diagram in Fig. 1 is as follows:

(1) $$\frac{dS}{dt}=b_{0}-(vp_{s}+d_{0})S-\beta (1-vp_{s})SI\;\frac{dE}{dt}=\beta (1-vp_{s})SI-(d_{1}+\alpha +(1-\alpha )vp_{e})E\;\frac{dI}{dt}=\alpha E-(d_{2}+\gamma +(1-\gamma )vp_{i})I\;\frac{dR}{dt}=vp_{s}S+vp_{e}(1-\alpha )E+(\gamma +(1-\gamma )vp_{i})I-d_{0}R$$

under the conditions that

(2) 0≤S(0),E(0),I(0),R(0)≤1.

The density S(t) at the time *t* is the faction of susceptible numbers, E(t),I(t) and R(t) are similar. The explanation of variables and parameters in (1) are presented in Table 1. A motivation for the model is that vaccination rate per day (*v*) cannot terminate the flow of the system immediately since the whole population cannot be vaccinated at once. A person can get vaccinated once he or she is susceptible, exposed, or infectious. In the first equation of System (1), the rate of change in susceptible depends on the numbers of vaccinated humans, $vp_{s}S$, and non-vaccinated humans, $(1-vp_{s})S$.

**Table 1:** Description of variables and parameters in the model.

Variable/Parameter	Interpretation
S	Fraction of susceptible cases
E	Fraction of exposed cases
I	Fraction of infectious cases
R	Fraction of recovered cases
*β*	Effective transmission rate of COVID-19
*α*	Changing rate from *E* to *I*
*γ*	Changing rate from *I* to *R*
*v*	Vaccination rate of population
*p*_*s*_	Effectiveness of vaccination in *S*
*p*_*e*_	Effectiveness of vaccination in *E*
*p*_*i*_	Effectiveness of vaccination in *I*
*b*_0_	Birth rate of population
*d*_0_	Death rate of population without COVID-19
*d*_1_	Death rate of exposed population plus *d*_0_
*d*_2_	Death rate of infectious population plus *d*_0_

Note that by the fundamental existence-uniqueness theorem for nonlinear systems, the nonlinear system (1) has a unique solution set (S(t),E(t),I(t),R(t)). To guarantee that the densities S(t),E(t),I(t), and R(t) in the model (1) are non-negative at any time t>0, we prove the following lemma.

Lemma 2.1 *If* (S,E,I,R) *is the continuous solution of the system* (1) *with initial* (2)*, then*

$$(S(t),E(t),I(t),R(t))\in {\left[0,\infty \right)}^{4}$$

*for any positive time* t>0*.*

Proof To prove this lemma we use the fact that a function *f* with f(0)≥0 is a non-negative function if $\frac{df}{dt}{|}_{t=t^{⁎}}\geq 0$ when $f(t^{⁎})=0$; i.e., the function *f* is non-decreasing at $t^{⁎}$. By the condition (2), there is $t_{s}$ such that S(t)≥0 on $0\leq t<t_{s}$ and $S(t_{s})=0$. Based on the first equation of Model (1), we have

$$\frac{dS}{dt}{|}_{t=t_{s}}=b_{0}>0$$

It implies that S(t)≥0 for any t≥0. Next, let $t_{i}$ be the time such that I(t)≥0 on $0\leq t<t_{i}$ and $I(t_{i})=0$. By the third equation of (1), we have

(3) $$\frac{dI}{dt}{|}_{t=t_{i}}=\alpha E(t_{i}).$$

Since *S* and *I* are non-negative on $\left[0,t_{i}\right]$, it follows by the second equation of (1) that

$$\frac{dE}{dt}+(d_{1}+\alpha +(1-\alpha )vp_{e})E\geq 0$$

on $\left[0,t_{i}\right]$. This implies that

(4) $$E(t_{i})\geq E(0)e^{-(d_{1}+\alpha +(1-\alpha )vp_{e})t_{i}}\geq 0$$

Equations (3) and (4) imply that $\frac{dI}{dt}{|}_{t=t_{i}}\geq 0$, so I(t)≥0 for any t≥0. It is easy to check that E(t)≥0 when I(t)≥0. Since *S*, *I*, and *E* are non-negative for t>0, it is obvious that R(t)≥0 for t≥0. □

From this lemma we can conclude that the set ${\left[0,\infty \right)}^{4}$ is positive invariant with respect to the model (1) and it attracts all solutions of the model.

### 2.1 Stability of equilibrium points

Equilibrium points of the system can be found by setting $\frac{dS}{dt}=0$, $\frac{dE}{dt}=0$, $\frac{dI}{dt}=0$, and $\frac{dR}{dt}=0$ of (1); i.e., solving the following system:

(5) $$0=b_{0}-(vp_{s}+d_{0})S-\beta (1-vp_{s})SI\;0=\beta (1-vp_{s})SI-(d_{1}+\alpha +(1-\alpha )vp_{e})E\;0=\alpha E-(d_{2}+\gamma +(1-\gamma )vp_{i})I\;0=vp_{s}S+vp_{e}(1-\alpha )E+(\gamma +(1-\gamma )vp_{i})I-d_{0}R$$

A disease-free equilibrium is an equilibrium when there is no spread of the disease; i.e., E≡0≡I. By solving (5), the disease-free equilibrium is unique in the form

(6) $$(S_{0},E_{0},I_{0},R_{0})=\left(\frac{b_{0}}{p_{s}v+d_{0}},0,0,\frac{b_{0}}{d_{0}}\right)$$

for fixed parameters $b_{0},p_{s},v$ and $d_{0}$.

Apart from the disease-free equilibrium, others equilibrium points (endemic equilibrium) of the model can be found by solving (5) under the conditions that S≢0,E≢0,I≢0 and R≢0. Since $(S(t),E(t),I(t),R(t))\in {\left[0,\infty \right)}^{4}$ as proved in Lemma 2.1, the endemic equilibrium is unique for fixed parameters of the model (1) and it is in the form

(7) $$\left(S_{1},E_{1},I_{1},R_{1}\right)$$

where

$$S_{1}=\frac{b_{0}}{vp_{s}+d_{0}+\beta (1-vp_{s})I_{1}}\;E_{1}=\frac{b_{0}-(vp_{s}+d_{0})S_{1}}{d_{1}+\alpha +(1-\alpha )vp_{e}}\;I_{1}=\frac{\alpha E_{1}}{d_{2}+\gamma +(1-\gamma )vp_{i}}\;R_{1}=\frac{b_{0}-d_{0}S_{1}-d_{1}E_{1}-d_{2}I_{1}}{d_{0}}$$

Next, let

(8) $$\left(S^{⁎},E^{⁎},I^{⁎},R^{⁎}\right)$$

be a representation of the equilibrium point in the form of (6) or (7). The following theorem describes stability of the equilibrium point (8).

Theorem 2.2 *For fixed parameters of the model* (1) *and the initial condition* (2)*, the equilibrium point of the model is locally asymptotic stable.*

Proof Consider the Jacobian matrix of the model (1) with respect to the equilibrium point (8) which is as follows:

(9) $$J=\left[\begin{array}{cccc} -vp_{s} & 0 & -AS^{⁎} & 0\\ AI^{⁎} & -(d_{1}+C_{\alpha }) & -AS^{⁎} & 0\\ 0 & \alpha & -(d_{2}+c_{\gamma }) & 0\\ vp_{s} & 8C_{\alpha }-\alpha & C_{\gamma } & -d_{0} \end{array}\right]$$

where $A=\beta (1-vp_{s})$, $C_{\alpha }=\alpha +(1-\alpha )vp_{e}$, and $C_{\gamma }=\gamma +(1-\gamma )vp_{i}$. The eigenvalues, *λ*, of the matrix (9) is computed by the equation det⁡(λI−J)=0; i.e., the eigenvalues are the solutions of the characteristic polynomial

(10) $$(d_{0}+\lambda )(D_{1}+D_{2}\lambda +D_{3}\lambda^{2}+\lambda^{3})=0$$

where

$$D_{1}=A^{3}I^{⁎}S^{⁎}+vp_{s}(C_{\alpha }C_{\gamma }+C_{\gamma }d_{1}+C_{\alpha }d_{2}+A^{2}S^{⁎})D_{2}=C_{\alpha }C_{\gamma }+C_{\gamma }d_{1}+C_{\alpha }d_{2}+d_{1}d_{2}+A^{2}S^{⁎}+vp_{s}(C_{\alpha }+C_{\gamma }+d_{1}+d_{2})D_{3}=C_{\alpha }+C_{\gamma }+d_{1}+d_{2}+vp_{s}.$$

It is obvious that $D_{1}>0$, $D_{2}>0$, and $D_{3}>0$. Since $D_{1},D_{2},D_{3}$ are positive real numbers, it follows that all solutions of Equation (10) have negative real parts. Therefore, the equilibrium point of the model (1) is locally asymptotic stable. □

### 2.2 The basic reproductive number and global stability

Using the matrices generation method [39], the basic reproductive number, $R_{0}$, is the dominant eigenvalue (the spectral radius) of $FV^{-1}$ where

(11) $$F=\left[\begin{array}{cc} 0 & \beta (1-vp_{s})S\\ \alpha & 0 \end{array}\right]$$

and

(12) $$V=\left[\begin{array}{cc} d_{1}+\alpha +(1-\alpha )vp_{e} & 0\\ 0 & d_{2}+\gamma +(1-\gamma )vp_{i} \end{array}\right].$$

Hence, the basic reproductive number, $R_{0}$, corresponding to the disease-free equilibrium (6) is in the form

(13) $$R_{0}=\sqrt{\frac{\alpha \beta (1-vp_{s})b_{0}}{\left(d_{1}+\alpha +(1-\alpha )vp_{e})(d_{2}+\gamma +(1-\gamma )vp_{i})(p_{s}v+d_{0}\right)}}$$

By (11) and (12), we note here that the dominant eigenvalues of $FV^{-1}$ and $V^{-1}F$ are the same. Based on this basic reproductive number ($R_{0}$), we then prove the following theorem about the global stability of the disease-free equilibrium (6).

Theorem 2.3 *If* $R_{0}<1$*, then the disease-free equilibrium* (6) *is globally asymptotic stable; on the other hand, the equilibrium is unstable if* $R_{0}>1$*.*

Proof Consider the matrix

$$u=\left[\begin{array}{cc} 1 & \frac{R_{0}(d_{2}+\gamma +(1-\gamma )vp_{i})}{\alpha } \end{array}\right]$$

where $d_{2},\gamma ,v$, and $p_{i}$ are parameters defined in Table 1. Note that *u* is a 1×2 matrix of positive real components. It is easy to check that

(14) $$u\left(R_{0}\left[\begin{array}{cc} 1 & 0\\ 0 & 1 \end{array}\right]-V^{-1}F\right)=0$$

where *F* and *V* are defined in (11) and (12), respectively. Equation (14) implies that

(15) $$uR_{0}=uV^{-1}F$$

Next, let

(16) $$X=\left[\begin{array}{c} E\\ I \end{array}\right].$$

We note here that X is a zero matrix only at the disease-free equilibrium. By using (1), we have

(17) $$\frac{dX}{dt}=\left[\begin{array}{c} \frac{dE}{dt}\\ \frac{dI}{dt} \end{array}\right]=\left[\begin{array}{cc} -(d_{1}+\alpha +(1-\alpha )vp_{e}) & \beta (1-vp_{s})S\\ \alpha & -(d_{2}+\gamma +(1-\gamma )vp_{i}) \end{array}\right]\left[\begin{array}{c} E\\ I \end{array}\right],=(F-V)X.$$

Define the Lyapunov function L as follows:

(18) $$L=uV^{-1}X$$

Since $uV^{-1}$ is a 1×2 matrix of positive real components and X is a non-negative matrix, it follows that L≥0 and we also have that L=0 if and only if E=0 and I=0. This implies that L is positive definite. Moreover, by (17) and (15) we obtain

$$\frac{dL}{dt}=uV^{-1}\frac{dX}{dt}=uV^{-1}(F-V)X=(uV^{-1}F-u)X=u(R_{0}-1)X.$$

Since $\frac{dL}{dt}<0$ if $R_{0}<1$, it follows that the disease-free equilibrium (6) is globally asymptotic stable [40]. On the other hand, if $R_{0}>1$, then $\frac{dL}{dt}>0$ which implies that the equilibrium is unstable. Note that in the case of $R_{0}=1$, we can conclude that the equilibrium is locally stable since $\frac{dL}{dt}=0$. □

## 3 Numerical simulations and interpretation of the model

We simulate the model (1) under two cases, Case I (US) and Case II (India) where the initial conditions and parameters are shown in Table 2. The simulation have been done by Mathematica program which approximates the solution of the model by the fourth order-Runge Kutta method (RK4).

**Table 2:** Parameter values and initial populations of US (Case I) and India (Case II) where initial susceptible *S*(0), infected *E*(0)+*I*(0), and recovered *R*(0) are based on data in [12], [41] last updated on November 1, 2020. The recovered rate is based on 14 days recovery with 96% recovered; i.e., $\gamma =\frac{1}{14}(0.96)$.

Initial/Parameter	Case I/Reference	Case II/Reference
S(0)	0.97286 [12]	0.994 [12]
E(0)+I(0)	0.00905 [41]	3.813 × 10^−4^[41]
R(0)	0.01809 [41]	5.569 × 10^−3^[41]
*β*	0.462 [42]	0.32 [43]
*α*	1/11.5 per day [22]	1/11.5 per day [22]
*γ*	0.0686 per day [12]	0.0686 per day [12]
*b*_0_	3.178 × 10^−5^ per day [44]	4.893 × 10^−5^ per day [45]
*d*_0_	2.377 × 10^−5^ per day [46]	1.992 × 10^−5^ per day [47]
*d*_1_	2.585 × 10^−5^ per day [12]	2.021 × 10^−5^ per day [41]
*d*_2_	2.585 × 10^−5^ per day [12]	2.021 × 10^−5^ per day [41]

The maximum reproductive number $R_{0}$ with respect to the disease-free equilibrium (6) occurs when there is zero vaccination (v=0); that is,

(19) $$R_{0}=\sqrt{\frac{\alpha \beta b_{0}}{d_{0}(d_{1}+\alpha )(d_{2}+\gamma )}}$$

**Figure 2:**
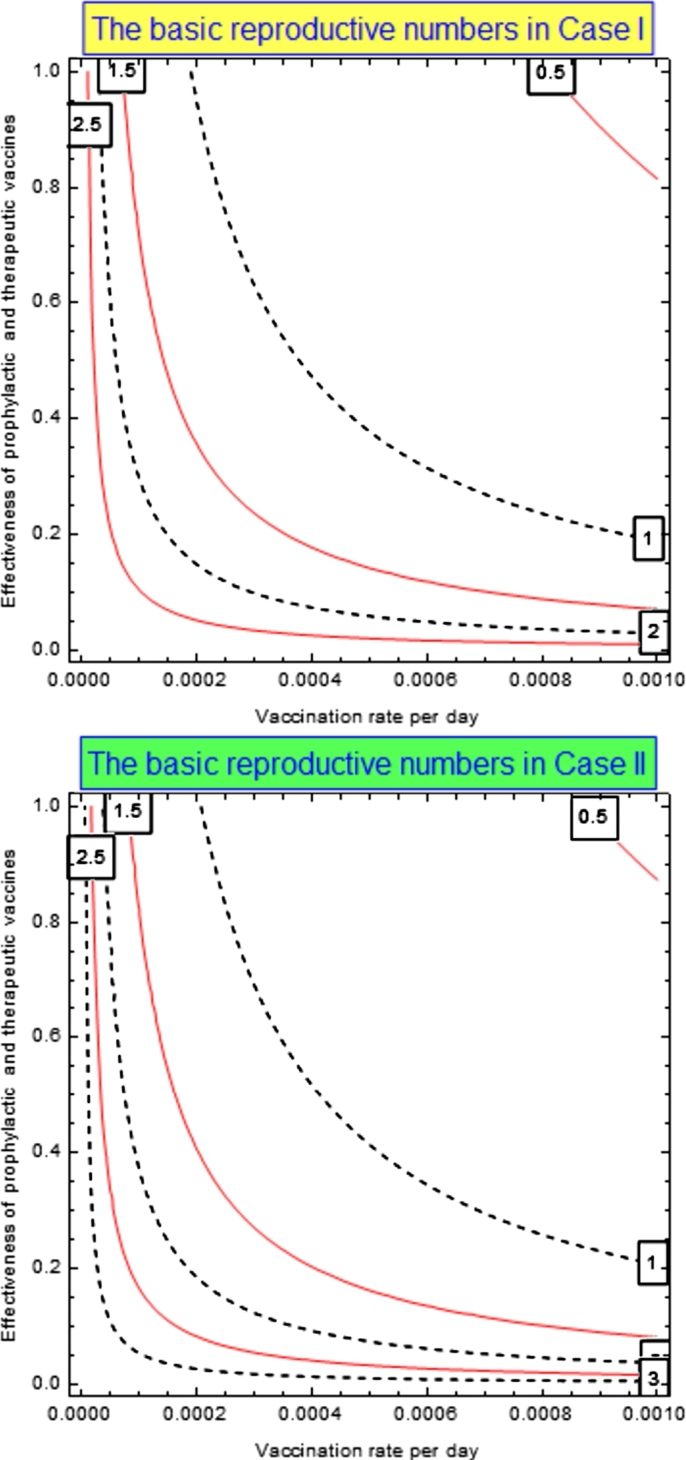
Contour plots of the reproductive numbers $R_{0}$ with 0 ≤ *v* ≤ 0.001 and 0 ≤ *p*_*s*_ = *p*_*e*_ = *p*_*i*_ ≤ 1 in US case (Case I) and India case (Case II).

Based on Table 2, the maximum $R_{0}$ of Case I is 2.99 and the maximum $R_{0}$ of Case II is 3.38. By increasing the vaccination rate (*v*), the values of $R_{0}$ are decreasing corresponding the effectiveness of prophylactic ($p_{s}$) and therapeutic ($p_{e},p_{i}$) vaccines, see Fig. 2. We note here as an example that $p_{s}=0.4$ means 40% effectiveness of prophylactic when applied to susceptible (*S*); i.e., if 100 people in *S* are administered a prophylactic vaccine, it will be 40 people recovered.

**Figure 3:**
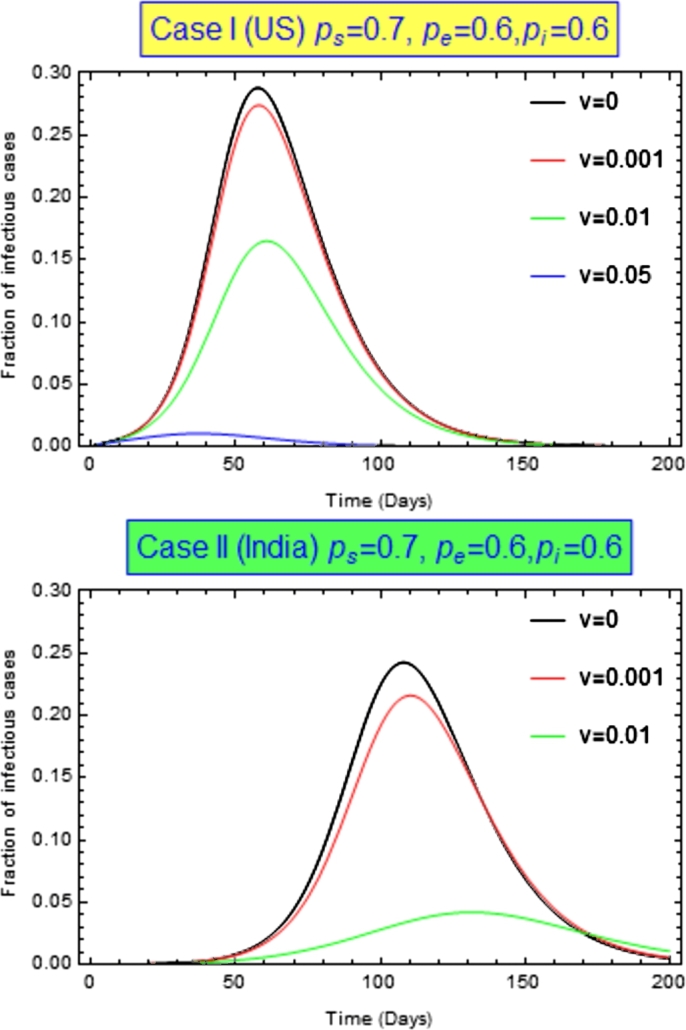
Fractions of US infectious cases and India infectious cases over time if we had 70% and 60% effectiveness of prophylactic and therapeutic vaccines, respectively, with different rates of vaccination, 0% (no vaccination process), 0.1%, 1%, and 5% (only US) per day of each population.

Fig. 2 shows that if the vaccination rate (*v*) is under 0.0002 or 0.02% per day of the populations, the basic reproductive numbers are still higher than 1 no matter how much vaccine's effectiveness is. If the rate of vaccination per day is increased (0-5% in the US and 0-1% in India), see Fig. 3, the infection rate in both countries will be decreased. This implies that the vaccination rate are play an important role to terminate the pandemic. However, the vaccine efficacy is also important, the effectiveness can vary the risk of illness [48]. SARS-CoV-2 is a zoonotic infection that has transmitted from a vertebrate to a human [49]. During outbreak, the virus infections in humans have been reported at higher rates than animals infections. SARS-CoV-2 may use animals and humans as reservoirs for reemerging, similar to SARS coronavirus [50], [51]. Thus, coronavirus disease may be a re-emerging viral diseases which is a diseases that has been observed previously within a population. To completely control SARS-CoV-2 infection, strategies for increasing vaccination rates is interesting to investigate for effective infection prevention and control of the disease. We need a sufficient vaccination rate depending on the power of vaccines and several doses of vaccine might be recommended. Based on Fig. 2, if we had a vaccine effectiveness higher than 20%, it would suffice to proceed 0.1% of the populations per day to reduce the basic reproductive numbers to be under 1. Moreover, with the same vaccine's efficiency of 70% of prophylactic and 60% of therapeutic, the US need higher rate of vaccination than India to flatten the curve as seen in Fig. 3.

The equilibrium point related to the US and India situations can be computed by using Equation (7). With the vaccination rate 0.1% per day of the US population (v=0.001) and 90% efficiency of prophylactic and therapeutic vaccines, the equilibrium point corresponding to the fixed parameters in Table 2 of the US case is $\left(S^{⁎},E^{⁎},I^{⁎},R^{⁎})=(0.0344,0,0,1.3026\right)$. If there is no vaccine, the equilibrium point of the US case is (0.1486,0.0003,0.0004,1.1876), that is the disease will not die out eventually. In the long term, there are about 0.04% infectious of the US population. India's case has $\left(S^{⁎},E^{⁎},I^{⁎},R^{⁎})=(0.0532,0,0,2.4032\right)$ for v=0.001 and 90% vaccines' efficiency and it has $\left(S^{⁎},E^{⁎},I^{⁎},R^{⁎})=(0.2145,0.0005,0.0006,2.2407\right)$ for no vaccines. Similarly to the US, a few percentages (0.06%) of India's population are infectious in the long term if there is no vaccine.

**Figure 4:**
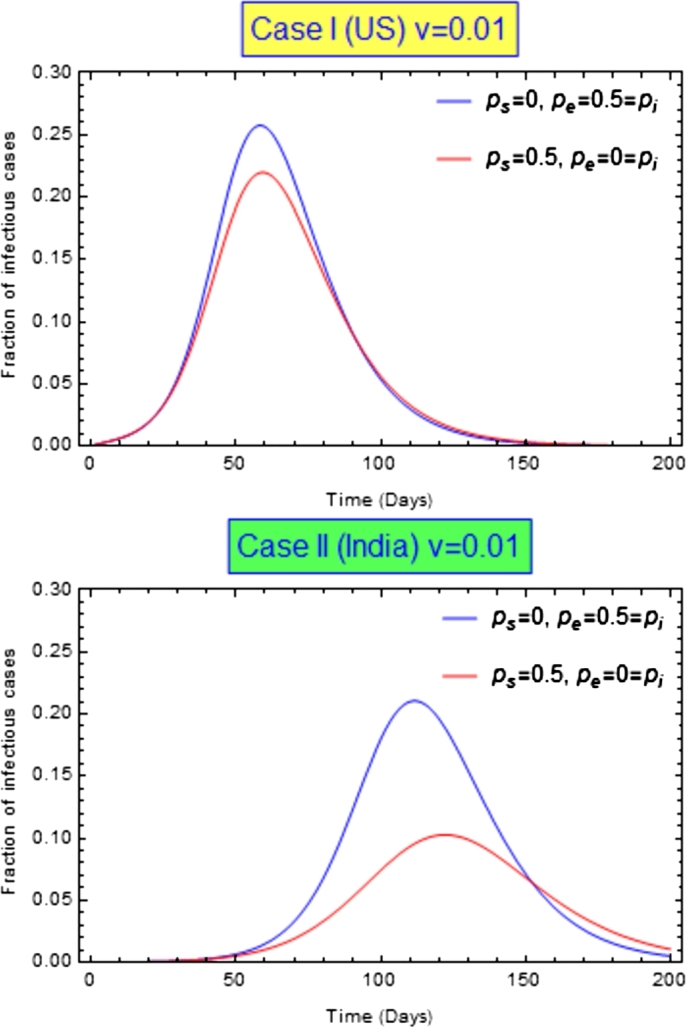
US and India cases when we have only prophylactic or only therapeutic with the same efficiency of the vaccines.

The difference between efficiency of prophylactic and therapeutic vaccines in human SARS-CoV-2 infection treatment is depicted as in Fig. 4. The effectiveness of both vaccines was set to the same values. The results showed that prophylactic vaccine has higher efficiency than therapeutic vaccine in both the US and India. Prophylactic vaccine will stimulate the immune system and then produce long-lived memory lymphocytes [52], [53]. Subsequently, the immune system can rapidly respond to virus infection, leading to a reduction of infected cases.

## 4 Conclusion

The constructed SEIR model in this manuscript gives a future perspective when we have a vaccine for COVID-19. The simulation shows that having an effective vaccine significantly flatten the peak of infectious population. The model illustrates that having a vaccine does not immediately terminate the pandemic. It takes a period of time depending on the effectiveness of the arrival vaccine and the vaccination rate. Under the same vaccine's effectiveness, the simulation shows that the US need vaccination rate per day higher than the rate used in India to achieve the same result. According to the formula of $R_{0}$ in Section 2, we see that the vaccination rate and the efficiency of vaccines play an important role to reduce the value of $R_{0}$. The theoretical results have confirmed that when the reproductive number $R_{0}$ of the pandemic is less than 1, the COVID-19 situation will be under control; i.e., the model is stable. The equilibrium point of the model for specific parameters gives the stationary flow of the pandemic situation in the long term that a few percentages of the considered populations will be infectious if we have no vaccine for COVID-19.

## Declarations

### Author contribution statement

P. Wintachai: Contributed reagents, materials, analysis tools or data; Wrote the paper.

K. Prathom: Conceived and designed the experiments; Performed the experiments; Analyzed and interpreted the data; Wrote the paper.

### Funding statement

This research did not receive any specific grant from funding agencies in the public, commercial, or not-for-profit sectors.

### Data availability statement

This work was supported by 10.13039/501100010034Walailak University grant no. WU64219.

### Declaration of interests statement

The authors declare no conflict of interest.

### Additional information

No additional information is available for this paper.
